# Bicuspid Aortic Valve: Genetic and Clinical Insights

**DOI:** 10.1055/s-0041-1730294

**Published:** 2021-12-03

**Authors:** Idit Tessler, Juliette Albuisson, Guillaume Goudot, Shai Carmi, Shoshana Shpitzen, Emmanuel Messas, Dan Gilon, Ronen Durst

**Affiliations:** 1 Department of Cardiology, Hadassah Medical Center, Jerusalem, Israel; 2 Faculty of Medicine, The Hebrew University, Jerusalem, Israel; 3 Braun School of Public Health and Community Medicine, The Hebrew University of Jerusalem, Jerusalem, Israel; 4 Oncogenetics laboratory, Centre George François Leclerc, Dijon, France; 5 Cardiovascular Department, Georges Pompidou European Hospital, Paris, France

**Keywords:** bicuspid aortic valve, genetics, congenital heart disease, thoracic aortic aneurysm, aortic dissection

## Abstract

Bicuspid aortic valve (BAV) is the most common valvular congenital heart disease, with a prevalence of 0.5 to 2% in the general population. Patients with BAV are at risk for developing cardiovascular complications, some of which are life-threatening. BAV has a wide spectrum of clinical presentations, ranging from silent malformation to severe and even fatal cardiac events. Despite the significant burden on both the patients and the health systems, data are limited regarding pathophysiology, risk factors, and genetics. Family studies indicate that BAV is highly heritable, with autosomal dominant inheritance, incomplete penetrance, variable expressivity, and male predominance. Owing to its complex genetic model, including high genetic heterogenicity, only a few genes were identified in association with BAV, while the majority of BAV genetics remains obscure. Here, we review the different forms of BAV and the current data regarding its genetics. Given the clear heritably of BAV with the potential high impact on clinical outcome, the clinical value and cost effectiveness of cascade screening are discussed.

## Introduction

Bicuspid aortic valve (BAV) is the most common valvular congenital heart disease, with a prevalence of 0.5 to 2% in the general population. 1 BAV was first described more than 500 years ago by Leonardo da Vinci, illustrating the valve anatomy. Since data on BAV clinical significance have been established, a substantial proportion of aortic valve diseases were found to be due to BAV, regardless of a patient's age. 2 Patients with BAV have an increased risk of developing aortic valve diseases such as calcification and stenosis, regurgitation, and infective endocarditis. Aortopathies are also prevalent among BAV patients. These include coarctation of the aorta, aortic aneurysm, and dissection. BAV patients are prone to require aortic valve replacement (AVR) and aortic surgery, procedures that carry substantial risks and costs. 3 Population-based studies have found a 53% risk for AVR and a 25% risk for aortic surgery during 25-year follow-up, and the risk for aortic dissection was eight times higher than in the general population. 4 Moreover, the mean age for valve replacement or surgical intervention for aortic dilation is markedly younger for BAV patients compared with patients with tricuspid aortic valve. 2 4 BAV was estimated to cause more morbidity and mortality than the combination of all other congenital heart defects, generating a considerable health burden to both patients and the health system. 5

**Fig. 1:**
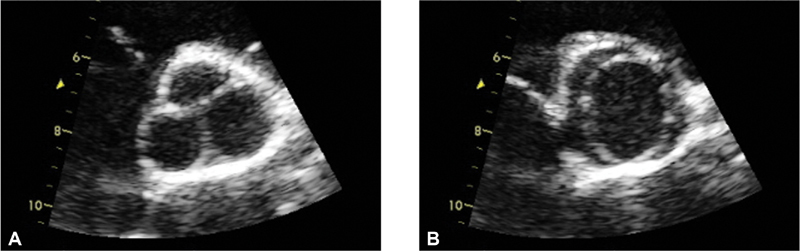
Transthoracic echocardiogram of bicuspid aortic valve (BAV), short axis view. ( **A** ) Diastolic image demonstrating a raphe that may mimic tricuspid valve. ( **B** ) Systolic image demonstrating only two leaflets with elliptical opening pattern. Morphology assessment of BAV must include systolic imaging, as diastolic imaging may be misleading. 40

BAV can be classified as sporadic BAV (sporadic isolated defect), familial nonsyndromic BAV (nsBAV; in clusters within families without associated anomaly), or syndromic BAV (considered familial and associated with other anomalies including cardiovascular defects). The method of choice for diagnosis and follow-up is echocardiography ( Fig. 1 ).

BAV clinical presentation varies significantly from a silent disease to severe life-threatening complications, even at a young age. Little is known about most dimensions of BAV, including the identity of the biochemical pathways involved in its pathogenesis. The determinants of the valve morphology and of the wide spectrum of clinical presentations and complications over time are mostly unelucidated.

Genetic data provide a very powerful and unbiased tool for understanding the basic mechanisms culminating in valve dysfunction and disease. Better understanding of the molecular processes of the disease may lead to future development of novel personalized management approaches, ultimately leading to individual risk stratification, sparing unnecessary interventions to low-risk patients, and preventing potentially fatal complications for patients at high risk.

Here, we summarize the current data regarding BAV genetics and discuss its potential clinical implication.

## Bicuspid Aortic Valve Genetics: Many Links Yet an Unsolved Riddle

**Fig. 2:**
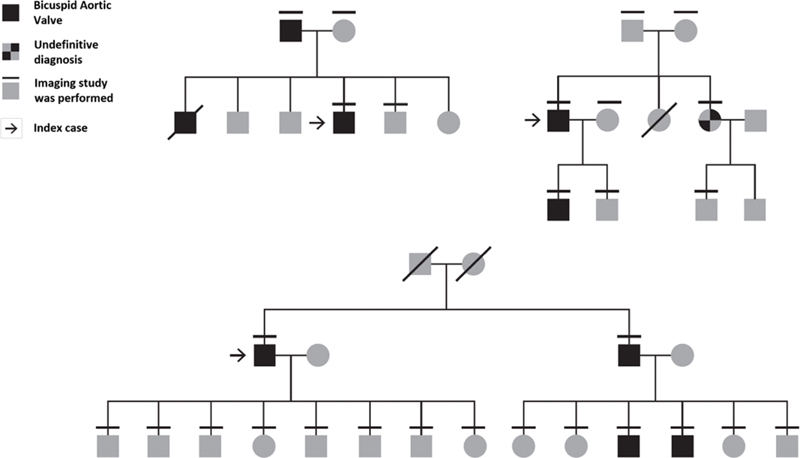
Examples of bicuspid aortic valve pedigrees, consistent with autosomal dominant inheritance, and low penetrance, as reflected by the limited number of clinically affected individuals. 40

**Fig. 3:**
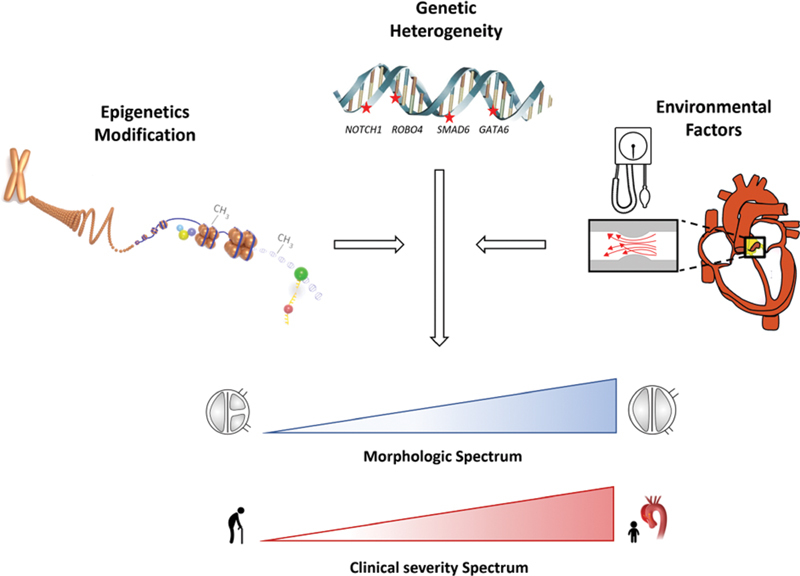
Illustration of the underling process in bicuspid aortic valve (BAV) development. Involvement of one or more genes is the primary insult. This might be modulated by epigenetic factors, such as chromatin modifications and DNA methylation affecting genetic regulatory elements. Environmental factors, such as longstanding abnormal blood flow and hypertension, may also contribute to BAV outcome. The epigenetics illustration was modified from the ENCODE portal (https://www.encodeproject.org/).

It is well established that BAV has a significant genetic component. 6 7 Various studies demonstrated familial clustering of BAV. 7 The prevalence of BAV was found to be 10-fold higher among first-degree relatives of an affected individual compared with the general population. 8 In family studies, the heritability index for BAV, representing the degree of phenotypic variance explained by inherited rather than environmental factors, was found to be as high as 89%, suggesting marked involvement of genetic factors on disease development. 7 Among familial BAV, most pedigrees suggest an autosomal-dominant inheritance pattern with incomplete penetrance and male predominance in a 3:1 ratio 8 ( Fig. 2 ). According to Mendelian genetics, autosomal-dominant inheritance pattern implies that half of first-degree relatives are expected to carry the disease-causing allele. Accounting for 50% penetrance (i.e., half of the carriers will demonstrate clinical disease), 25% of first-degree relatives are expected to be clinically affected with BAV. However, the actual rate of BAV among first degree relatives in family studies ranges from 6 to 30%. This large range, along with the wide spectrum of structural and clinical phenotypes, is thought to be the result of the complexity of the developmental mechanisms at play in aortic valve development, involving genetic, epigenetic, and environmental factors ( Fig. 3 ).

A high prevalence rate of aorthopathies, including aneurysm, dissection, and aortic coarctation, has been demonstrated among BAV patients and their relatives. Both the aortic root and the aortic valve have the same specific embryologic origin: the cardiac neural crest and the second heart field. 9 Thoracic aortic aneurysm (TAA) frequently affects patients with BAV, or their first-degree relatives with a morphologically normal valve. TAA and BAV are thus thought to have a common genetic etiology. 6 This observation adds support to the concept that BAV does not represent a dichotomous phenotype but would rather be integrated in a continuous spectrum of phenotypic expressions.

### Nonsyndromic Bicuspid Aortic Valve Genetics

Since 2005, with the identification of *NOTCH1* in nsBAV cases, few other genes were found to be associated with nsBAV with varying degrees of supporting evidence ( Table 1 ). Each of these genes explains only a small percentage of the overall nsBAV prevalence and involves different molecular pathways that do not necessarily assemble into one common mechanism. In light of its high phenotypic and genotypic heterogeneity, establishing a genetic causality for BAV is challenging. Causality can only be determined when the mutation has a robust effect, the familial segregation and linkage analyses are strong, and when the association is supported by experimental and functional models. 10

**Table 1:** Main genes associated with bicuspid aortic valve

Main genes associated with BAV in humans, and the methodology used in each study for gene identification		Main genes associated with BAV in mice, with the percentage of mice that devloped BAV	
Humans genes	Genetic approach	Mouse genes	Prevalence of BAV (%)
*NOTCH1* 11	Linkage analysis	*Acvr1/Alk1* 36	78–83
*GATA4* 19	Genome-wide association study	*Gata5* 18	25
*GATA5* 20	Target gene sequencing	*Gata6* 46	25
*GATA6* 21	Family study	*Matr3* 47	12
*NKX2–5* 23	Family study	*Nkx2–5* 35	2–20
*TBX20* 14	Copy number variation analysis	*Nos3* 32	42
*SMAD6* 13	Candidate gene resequencing	*Robo1/Robo2* 33	100
*ROBO4* 24	Family study (whole exome sequencing)	*Robo4* 24	15

Abbreviation: BAV, bicuspid aortic valve.

**Fig. 4:**
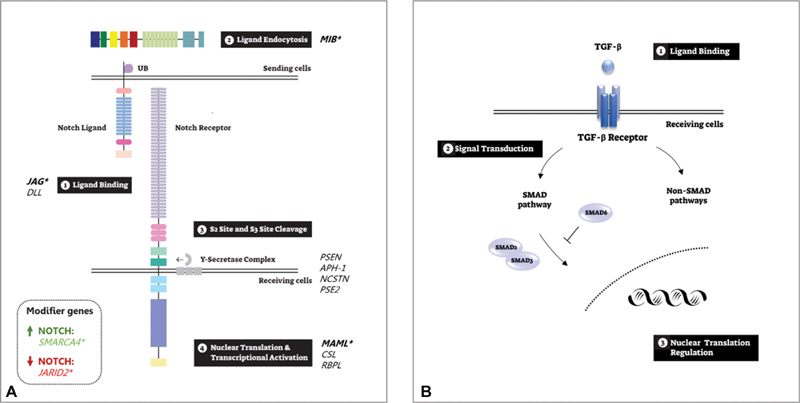
Bicuspid aortic valve (BAV)–associated pathways. ( **A** ) *NOTCH* pathway. A graphical representation of NOTCH pathway activation: (1) NOTCH receptor extracellular domain binds to its ligand's extracellular domain; (2) Notch ligand ubiquitination allowing endocytosis of the ligand in the signal-sending cell; (3) then, the notch receptor undergoes sequential proteolytic cleavages that result in the release of the Notch intracellular domain (NICD) and the notch extracellular domain; and (4) the NICD translocates to the nucleus and acts as a transcriptional regulator. *Genes associated with BAV: *MIB1* KO mice developed; JAG1 was associated with BAV in family and mice studies; Variants in *SMARCA4* , *JARID2* and *MAML* were identified in familial BAV. ( **B** ) *TGF-β* signaling pathway. The interaction between the *TGF-β* signaling pathway and the SMAD proteins: (1) *TGF-β* binding to its receptors triggering the signaling activation in the receiving cell; among others modifiers, the signal transduction is regulated by the SMAD proteins, while SMAD6 functions as the negative regulator; and (3) nuclear regulation affects cell proliferation, differentiation and growth. *JARID2* , Jumonji and AT-rich interaction domain containing 2; *MAML* , mastermind-like transcriptional coactivator; *MIB1* , mindbomb 1 *SMARCA4* , SWI/SNF-related, matrix-associated, actin-dependent regulator of chromatin, subfamily A, member 4; *TGF-β* , transforming growth factor beta 1. Image modified from Guo et al. 48

*NOTCH* pathway: the first and currently single gene considered definitively causal for nsBAV is *NOTCH1* . 11 *NOTCH1* signaling is a highly conserved pathway of signal transduction, leading to transcription of endothelial and vascular smooth muscle cells. Altered *NOTCH* signaling is a well-known cause of human cardiovascular disease. *NOTCH1* genetic variants were demonstrated to be associated with the development of calcific aortic valve stenosis, with or without BAV. Yet, this gene is estimated to be involved in only approximately 5 to 10% of nsBAV cases, leaving the vast majority of the genetic causes of BAV unexplained. Other members of the *NOTCH1* pathway ( Fig. 4 ) were linked to BAV and to other left-ventricular outflow tract obstruction pathologies, including mastermind-like transcriptional coactivator 1 ( *MAML1* ), rho GTPase activating protein 31 ( *ARHGAP31* ), jumonji and AT-rich interaction domain containing 2 ( *JARID2* ), and SWI/SNF-related matrix-associated actin-dependent regulator of chromatin, subfamily A, member 4 ( *SMARCA4* ). 12

*TGF* - *β* Pathway: The *SMAD* family member 6 ( *SMAD6* ) gene encodes a signal transduction protein highly expressed in the embryonic heart and involved in many pathways, including transforming growth factor beta ( *TGF-β* ). This pathway plays a key role in vascular matrix remolding and was linked to connective tissue disorders ( Fig. 4 ). The association of *SMAD6* with BAV was shown by targeted resequencing of individuals with BAV and TAA, contributing to the development of BAV/TAA in 2.5% of the cases. 13 Recently, T-box transcription factor 20 ( *TBX20* ) was identified as a possible contributing gene for BAV using copy number variation analysis, explaining 1% of the BAV/TAA cases. 14 This gene was found to be related to *SMAD6* in in-vivo studies, and was described in association to other congenital cardiac malformations. 15 Of note, other components of the TGFβ pathway ( *TGFβ2* , *TGF* - *β3* , *TGF* - *ßR1* , *TGF* - *ßR2* , and *SMAD3* ) are involved in syndromic aortopathies where BAV is present in 5 to 30% of the cases. 16 17

The *GATA* family: *GATA* binding protein genes encode zinc-finger transcription factors that play a role in heart valve differentiation. 18 *GATA4* was recently identified as a predisposing gene for BAV in a human genome-wide association study (GWAS) involving 466 BAV cases and 4,660 controls, with odds ratios ranging from 1.4 to 2.4 depending on the variant. 19 Rare variants of the *GATA5* gene, highly expressed in the endocardium, were also linked to nsBAV, 20 although these results have not been consolidated in subsequent studies. A *GATA6* disruptive variant was found in an nsBAV family, 21 and in vitro studies demonstrated that *GATA6* haploinsufficiency interrupts the aortic valve remodeling and extracellular matrix composition. 22 Loss-of-function mutations in the *NK2 Homeobox 5* ( *NKX2.5* ) gene, which encodes a homeodomain-containing transcription factor that is involved in the aortic valve development, was found in a nsBAV family to disrupt the interaction between *NKX2.5* and *GATA5* , supporting involvement of both genes in the pathology. 23

The roundabout guidance receptor 4 ( *ROBO4* ) gene is involved in endothelial function. Rare variants in the gene were identified by whole exome sequencing in a BAV/TAA family study. 24

Genetic loci linked to BAV: linkage analyses demonstrated the involvement of human chromosomal regions 18q, 5q, and 13q in BAV alone, and between BAV/TAA and human chromosomal regions 15q25–26, 25 suggesting that unelucidated genetic defects remain to be investigated.

### Syndromic Bicuspid Aortic Valve Genetics

BAV can be syndromic, that is, presenting within a constellation of cardiac and noncardiac anomalies ( Table 2 ). The highest occurrence of BAV is found in Turner's syndrome. Turner's syndrome results from complete or partial missing of one X chromosome (45X). This leads to a complex developmental disorder, including cardiovascular anomalies. BAV occurs in 15 to 30% of patients and often coexists with coarctation of the aorta. 26 The high prevalence of BAV in Turner's syndrome may be related to high diagnostic rate due to routine cardiac imaging performed in these patients, but may also suggest X-chromosome involvement in BAV formation. This is also supported by the 3:1 male predominance found in BAV, leading to the hypothesis that X chromosome gene hemizygosity (i.e., having one copy only) is involved in BAV development.

**Table 2:** The main syndromes which may present with bicuspid aortic valve (BAV), their genetic origin, and the prevalence of BAV within each syndrome

Syndrome	Genetic origin	Prevalence of BAV (%)
Turner's syndrome	Monosomy X	15–30
Marfan's syndrome	*FBN1*	1.8
Loeys–Dietz syndromes	TGF-β pathway	10–30
Shone's complex	*NOTCH1*	50
Andersen's syndrome	*KCNJ2*	10 a

a A total of 10% genotype-positive family member presented with BAV.

Marfan's syndrome (MFS) is a rather common connective tissue disorder manifesting by aortic root dilation among other phenomena. BAV was initially considered more prevalent than in the general population. 27 A recent larger study that included more than 1,400 MFS case, has demonstrated that the prevalence of BAV was 1.8%, equivalent to the population prevalence. 28 However, BAV presentation in MFS was associated with a more severe aortic aneurysm phenotype necessitation repair at an earlier age. 27

Loeys–Dietz syndromes are a group of connective tissue disorders close to MFS. These syndromic aortopathies are the consequence of abnormal TGF-β signaling, and association with BAV was demonstrated. 17

As illustrated here, the frequent cooccurrence of BAV and aortic aneurysms in nsBAV and in sporadic BAV, is also the rule in syndromic BAV, supporting the hypothesis that disruption of connective tissue homeostasis is related with BAV.

BAV was also described in Shone complex, a syndrome of multiple left heart obstructive lesions. Like nsBAV, it was also associated with *NOTCH1* mutations. 29 BAV is also present in other systemic disorders, such as DiGeorge's syndrome (22q11 deletion), Down's syndrome, and Andersen's syndrome, at very lower frequency. The malformation is also reported in association with other isolated cardiovascular disorders 30 including hypoplastic left heart syndrome, coarctation of the aorta, ventricular septal defects, patent ductus arteriosus, and atrial septal defects. 31

## Animal Models

Animal models may serve as an additional approach for understanding BAV genetics and pathophysiology. There are several mouse and Syrian hamster models for BAV, some of which were developed to support candidate genes found in humans. Of note, similarly to family studies in humans, all animal models have demonstrated incomplete penetrance and, in most cases, presented with other cardiac malformations. In some, male predominance was also observed. 32 The main human and mouse genes involved in BAV are listed in Table 1 .

*Notch1* knockout (KO) mice die from cardiac malformation. These mice developed severe aortic valve calcification. Disruption of the Robo signaling pathway ( *Robo1* and *Robo2* ) in transgenic mice led to BAV development. 33 This pathway was shown to play a role in Notch regulation and was also associated with BAV in humans. The Gata gene family ( *Gata4, Gata5,* and *Gata6* ) was linked to BAV in mice, as well as in humans, leading to BAV with variable penetrance ( Table 1 ). Nitric oxide synthase ( *Nos* ) produces nitric oxide (NO) that has an important role in cell growth and apoptosis. Mice with induced endothelial NOS-deficiency demonstrated abnormal aortic valve development including BAV. 32 A significantly reduced expression of NOS protein was demonstrated in aortic endothelial cells from BAV patients as compared with normal valve controls. 34 *Nkx2–5* KO mice developed BAV among other septal and valvular malformations. 35 This gene is a notable example of a pleiotropic genetic effect (in which one gene leads to more than one phenotype). 35 Notably, a pleiotropic effect is far more often the rule than the exception in many congenital heart disease genes. Tissue-specific KO of activin A receptor type 1 ( *Acvr1* ) resulted in aortic valve disorders including BAV, supporting the gene's role in valvular development as seen in TAAD/BAV human cases. 36 However, there is not a full correspondence between the genetic landscapes in mice and in humans. As an example, *ROBO4* has been involved in BAV/TAA in humans at heterozygous state with high penetrance, but the effect of complete (homozygous) *Robo4* loss of function in mice shows a very low penetrance (15%) with a variety of aortic valve defects. 24

## Bicuspid Aortic Valve Phenotypes: Sievers Classification

Anatomically the bicuspid valve morphology phenotypes are classified according to the cusp's fusion. A “pure” form of BAV consists of two cusps of equal size with no raphe between them and is relativity rare, while the more common configuration of bicuspid valve consists of two unequal cusps, the larger one characterized by a raphe formed between the two fused cusps. A rare form of unicuspidal valve is also found. The BAV morphology type is usually defined by echocardiography and is classified according to Sievers classification as follows: type 0 (no raphe); type 1 (one raphe) with subtypes (1) 1 LR for left–right coronary cusps fusion, (2) 1 RN for right and noncoronary, and (3) 1 NL for noncoronary and left coronary cusps; and type 2 (two raphes). Each morphology type is associated with different pathologies of the valve and the aorta, and may even affect prognosis. 37 It was hypothesized that the different types developed from distinct embryological origins. 38 Our data, as well as previously published data, show, in a large set of pedigrees, that different BAV types are present in a family. 39 40 To date, no correlation between Sievers type BAV and genetic status was demonstrated. This, once again, highlights the complexity of BAV genetics and phenotypic variability.

## Cascade Screening: What Is the Clinical Utility?

High heritability of BAV raises the question of “cascade screening” of relatives of a BAV case. Cascade screening is a method to identify individuals at risk for a genetic condition by the process of systematic screening of first-degree relatives of the index case. The 2014 American Heart Association/American College of Cardiology (AHA/ACC) valvular heart disease guidelines recommend clinical screening of first-degree relatives only if the patient with BAV has an associated aortopathy or a family history of valvular heart disease or aortopathy. 41 There is no clear recommendation, however, for screening in patients with noncomplicated BAV. The European Society of Cardiology and the European Association for Cardio-Thoracic Surgery guidelines for the management of valvular heart disease consider BAV as a risk factor for aortic regurgitation and suggest echocardiographic screening of first-degree relatives. 42 The Canadian Cardiovascular Society indicated screening by echocardiography of first-degree relatives of bicuspid patients, including screening of family members in the pediatric age range. 43 The screening examination by echocardiography itself does not involve any risk for the patient. It can detect BAV or associated pathologies at an early stage and hence prevent complications. This, however, may come at a considerable emotional burden to the families. As described above, 6 to 30% of first-degree relatives are expected to have BAV or related anomaly. It is currently not clear how many of these will clinically benefit from familial screening. Cost analysis studies have demonstrated a significant cost-effectiveness for echocardiography screening. 44 45 Additional studies are needed to establish the best terms and timing of the optimal screening program.

## Conclusion

Even after more than 500 years of its first description by Leonardo da Vinci, BAV still poses a great challenge to clinicians. It presents with a wide clinical and structural phenotypic spectrum, from a silent malformation to a severe complicated disease with significant morbidity and mortality. Biological research to understand BAV and its cause, as in other cardiac malformations, is very active since the discovery of *NOTCH1* role in BAV. Complimentary genetic approaches, including association, linkage, and candidate-gene studies, have allowed identification of few other genes, accounting for only a small fraction of the genetic weight in the disease, and our understanding remains very limited. This is probably explained by a complex developmental process. Epigenetic and microenvironmental factors might weight more significantly than expected, unveiling complex inheritance including polygenic involvement. Deciphering genetic models of BAV is now the new challenge, aiming at the objective of optimizing patient's risk stratification and clinical management according to the individual risk.

## References

[1] Braverman A C The bicuspid aortic valve and associated aortic disease 4th ed. Philadelphia, PA Saunders/Elsevier 2013 179 198

[2] Roberts W C Ko J M Frequency by decades of unicuspid, bicuspid, and tricuspid aortic valves in adults having isolated aortic valve replacement for aortic stenosis, with or without associated aortic regurgitation Circulation 2005 111 07 920 925 15710758 10.1161/01.CIR.0000155623.48408.C5

[3] Tzemos N Therrien J Yip J Outcomes in adults with bicuspid aortic valves JAMA 2008 300 11 1317 1325 18799444 10.1001/jama.300.11.1317

[4] Michelena H I Khanna A D Mahoney D Incidence of aortic complications in patients with bicuspid aortic valves JAMA 2011 306 10 1104 1112 21917581 10.1001/jama.2011.1286

[5] Ward C Clinical significance of the bicuspid aortic valve Heart 2000 83 01 81 85 10618341 10.1136/heart.83.1.81 PMC1729267

[6] Loscalzo M L Goh D LM Loeys B Kent K C Spevak P JDH Dietz H C Familial thoracic aortic dilation and bicommissural aortic valve: a prospective analysis of natural history and inheritance Am J Med Genet A 2007 143A 17 1960 1967 17676603 10.1002/ajmg.a.31872

[7] Cripe L Andelfinger G Martin L J Shooner K Benson D W Bicuspid aortic valve is heritable J Am Coll Cardiol 2004 44 01 138 143 15234422 10.1016/j.jacc.2004.03.050

[8] Siu S C Silversides C K Bicuspid aortic valve disease J Am Coll Cardiol 2010 55 25 2789 2800 20579534 10.1016/j.jacc.2009.12.068

[9] Martin P S Kloesel B Norris R A Lindsay M Milan D Body S C Embryonic development of the bicuspid aortic valve J Cardiovasc Dev Dis 2015 2 04 248 272 28529942 10.3390/jcdd2040248 PMC5438177

[10] Marian A J Causality in genetics: the gradient of genetic effects and back to Koch's postulates of causality Circ Res 2014 114 02 e18 e21 24436434 10.1161/CIRCRESAHA.114.302904 PMC3896867

[11] Garg V Muth A N Ransom J F Mutations in NOTCH1 cause aortic valve disease Nature 2005 437(7056):270 274 16025100 10.1038/nature03940

[12] MIBAVA Leducq consortium Preuss C Capredon M Wünnemann F Family based whole exome sequencing reveals the multifaceted role of notch signaling in congenital heart disease PLoS Genet 2016 12 10 e1006335 27760138 10.1371/journal.pgen.1006335 PMC5070860

[13] Mibava Leducq Consortium Gillis E Kumar A A Luyckx I Candidate gene resequencing in a large bicuspid aortic valve-associated thoracic aortic aneurysm cohort: *SMAD6* as an important contributor Front Physiol 2017 8 400 28659821 10.3389/fphys.2017.00400 PMC5469151

[14] MIBAVA Leducq Consortium Luyckx I Kumar A A Reyniers E Copy number variation analysis in bicuspid aortic valve-related aortopathy identifies TBX20 as a contributing gene Eur J Hum Genet 2019 27 07 1033 1043 30820038 10.1038/s41431-019-0364-y PMC6777542

[15] de Pater E Ciampricotti M Priller F Bmp signaling exerts opposite effects on cardiac differentiation Circ Res 2012 110 04 578 587 22247485 10.1161/CIRCRESAHA.111.261172 PMC4924880

[16] van de Laar I MBH van der Linde D Oei E HG Phenotypic spectrum of the SMAD3-related aneurysms-osteoarthritis syndrome J Med Genet 2012 49 01 47 57 22167769 10.1136/jmedgenet-2011-100382

[17] Bertoli-Avella A M Gillis E Morisaki H Mutations in a TGF-β ligand, TGFB3, cause syndromic aortic aneurysms and dissections J Am Coll Cardiol 2015 65 13 1324 1336 25835445 10.1016/j.jacc.2015.01.040 PMC4380321

[18] Laforest B Andelfinger G Nemer M Loss of Gata5 in mice leads to bicuspid aortic valve J Clin Invest 2011 121 07 2876 2887 21633169 10.1172/JCI44555 PMC3223824

[19] Yang B Zhou W Jiao J Protein-altering and regulatory genetic variants near GATA4 implicated in bicuspid aortic valve Nat Commun 2017 8 15481 28541271 10.1038/ncomms15481 PMC5458508

[20] Padang R Bagnall R D Richmond D R Bannon P G Semsarian C Rare non-synonymous variations in the transcriptional activation domains of GATA5 in bicuspid aortic valve disease J Mol Cell Cardiol 2012 53 02 277 281 22641149 10.1016/j.yjmcc.2012.05.009

[21] Xu Y-J Di R-M Qiao Q GATA6 loss-of-function mutation contributes to congenital bicuspid aortic valve Gene 2018 663 115 120 29653232 10.1016/j.gene.2018.04.018

[22] Bicuspid Aortic Valve Consortium Gharibeh L Komati H Bossé Y GATA6 regulates aortic valve remodeling, and its haploinsufficiency leads to right-left type bicuspid aortic valve Circulation 2018 138 10 1025 1038 29567669 10.1161/CIRCULATIONAHA.117.029506 PMC6151169

[23] Qu X-K Qiu X-B Yuan F A novel NKX2.5 loss-of-function mutation associated with congenital bicuspid aortic valve Am J Cardiol 2014 114 12 1891 1895 25438918 10.1016/j.amjcard.2014.09.028

[24] Baylor-Hopkins Center for Mendelian Genomics MIBAVA Leducq Consortium Gould R A Aziz H Woods C E ROBO4 variants predispose individuals to bicuspid aortic valve and thoracic aortic aneurysm Nat Genet 2019 51 01 42 50 30455415 10.1038/s41588-018-0265-y PMC6309588

[25] Martin L J Ramachandran V Cripe L H Evidence in favor of linkage to human chromosomal regions 18q, 5q and 13q for bicuspid aortic valve and associated cardiovascular malformations Hum Genet 2007 121 02 275 284 17203300 10.1007/s00439-006-0316-9

[26] Mortensen K H Andersen N H Gravholt C H Cardiovascular phenotype in Turner syndrome--integrating cardiology, genetics, and endocrinology Endocr Rev 2012 33 05 677 714 22707402 10.1210/er.2011-1059

[27] Nistri S Porciani M C Attanasio M Abbate R Gensini G F Pepe G Association of Marfan syndrome and bicuspid aortic valve: frequency and outcome Int J Cardiol 2012 155 02 324 325 22225761 10.1016/j.ijcard.2011.12.009

[28] Milleron O Ropers J Arnoult F Clinical significance of aortic root modification associated with bicuspid aortic valve in Marfan syndrome Circ Cardiovasc Imaging 2019 12 03 e008129 30841707 10.1161/CIRCIMAGING.118.008129

[29] Escárcega R O Michelena H I Bove A A Bicuspid aortic valve: a neglected feature of Shone's complex? Pediatr Cardiol 2014 35 01 186 187 24078196 10.1007/s00246-013-0804-3

[30] Andelfinger G Tapper A R Welch R C Vanoye C G George A LJ Jr. Benson D W KCNJ2 mutation results in Andersen syndrome with sex-specific cardiac and skeletal muscle phenotypes Am J Hum Genet 2002 71 03 663 668 12148092 10.1086/342360 PMC379203

[31] BAVCon Investigators Prakash S K Bossé Y Muehlschlegel J D A roadmap to investigate the genetic basis of bicuspid aortic valve and its complications: insights from the International BAVCon (Bicuspid Aortic Valve Consortium) J Am Coll Cardiol 2014 64 08 832 839 25145529 10.1016/j.jacc.2014.04.073 PMC4485610

[32] Lee T C Zhao Y D Courtman D W Stewart D J Abnormal aortic valve development in mice lacking endothelial nitric oxide synthase Circulation 2000 101 20 2345 2348 10821808 10.1161/01.cir.101.20.2345

[33] Mommersteeg M TM Yeh M L Parnavelas J G Andrews W D Disrupted Slit-Robo signalling results in membranous ventricular septum defects and bicuspid aortic valves Cardiovasc Res 2015 106 01 55 66 25691540 10.1093/cvr/cvv040 PMC4362403

[34] Aicher D Urbich C Zeiher A Dimmeler S Schäfers H-J Endothelial nitric oxide synthase in bicuspid aortic valve disease Ann Thorac Surg 2007 83 04 1290 1294 17383329 10.1016/j.athoracsur.2006.11.086

[35] Biben C Weber R Kesteven S Cardiac septal and valvular dysmorphogenesis in mice heterozygous for mutations in the homeobox gene Nkx2-5 Circ Res 2000 87 10 888 895 11073884 10.1161/01.res.87.10.888

[36] Thomas P S Sridurongrit S Ruiz-Lozano P Kaartinen V Deficient signaling via Alk2 (Acvr1) leads to bicuspid aortic valve development PLoS One 2012 7 04 e35539 22536403 10.1371/journal.pone.0035539 PMC3334911

[37] Schaefer B M Lewin M B Stout K K The bicuspid aortic valve: an integrated phenotypic classification of leaflet morphology and aortic root shape Heart 2008 94 12 1634 1638 18308868 10.1136/hrt.2007.132092

[38] Fernández B Durán A C Fernández-Gallego T Bicuspid aortic valves with different spatial orientations of the leaflets are distinct etiological entities J Am Coll Cardiol 2009 54 24 2312 2318 19958967 10.1016/j.jacc.2009.07.044

[39] Robledo-Carmona J Rodríguez-Bailón I Carrasco-Chinchilla F Hereditary patterns of bicuspid aortic valve in a hundred families Int J Cardiol 2013 168 04 3443 3449 23684596 10.1016/j.ijcard.2013.04.180

[40] Tessler I Goudot G Albuisson J Is bicuspid aortic valve, morphology genetically determined? A family-based study Am J Cardiol 2021(e-pub ahead of print) 10.1016/j.amjcard.2021.09.051 34799086

[41] Nishimura R A Otto C M Bonow R O 2014 AHA/ACC guideline for the management of patients with valvular heart disease: A report of the American college of cardiology/American heart association task force on practice guidelines Circulation 2014 129 23 2440 2492 24589852 10.1161/CIR.0000000000000029

[42] ESC Scientific Document Group Baumgartner H Falk V Bax J J 2017 ESC/EACTS Guidelines for the management of valvular heart disease Eur Heart J 2017 38 36 2739 2791 28886619 10.1093/eurheartj/ehx391

[43] Canadian Cardiovascular Society Boodhwani M Andelfinger G Leipsic J Canadian Cardiovascular Society position statement on the management of thoracic aortic disease Can J Cardiol 2014 30 06 577 589 24882528 10.1016/j.cjca.2014.02.018

[44] Tessler I Leshno M Shmueli A Shpitzen S Durst R Gilon D Cost-effectiveness analysis of screening for first-degree relatives of patients with bicuspid aortic valve Eur Heart J Qual Care Clin Outcomes 2021 7 05 447 457 34227670 10.1093/ehjqcco/qcab047

[45] Hales A R Mahle WT Echocardiography screening of siblings of children with bicuspid aortic valve Pediatrics 2014 133 05 e1212 e1217 24709923 10.1542/peds.2013-3051

[46] Laforest B Nemer M GATA5 interacts with GATA4 and GATA6 in outflow tract development Dev Biol 2011 358 02 368 378 21839733 10.1016/j.ydbio.2011.07.037

[47] Quintero-Rivera F Xi Q J Keppler-Noreuil K M MATR3 disruption in human and mouse associated with bicuspid aortic valve, aortic coarctation and patent ductus arteriosus Hum Mol Genet 2015 24 08 2375 2389 25574029 10.1093/hmg/ddv004 PMC4380077

[48] Guo B McMillan B J Blacklow S C Structure and function of the Mind bomb E3 ligase in the context of Notch signal transduction Curr Opin Struct Biol 2016 41 38 45 27285058 10.1016/j.sbi.2016.05.012 PMC5143217

